# Brain subnetworks most sensitive to alterations of functional connectivity in Schizophrenia: a data-driven approach

**DOI:** 10.3389/fninf.2023.1175886

**Published:** 2023-05-18

**Authors:** Farzaneh Keyvanfard, Alireza Rahimi Nasab, Abbas Nasiraei-Moghaddam

**Affiliations:** ^1^School of Cognitive Sciences, Institute for Research in Fundamental Sciences (IPM), Tehran, Iran; ^2^Biomedical Engineering Department, Amirkabir University of Technology, Tehran, Iran

**Keywords:** blind analysis, functional connectivity, rs-fMRI, independent component analysis, ICA, brain subnetworks, schizophrenia

## Abstract

Functional connectivity (FC) of the brain changes in various brain disorders. Its complexity, however, makes it difficult to obtain a systematic understanding of these alterations, especially when they are found individually and through hypothesis-based methods. It would be easier if the variety of brain connectivity alterations is extracted through data-driven approaches and expressed as variation modules (subnetworks). In the present study, we modified a blind approach to determine inter-group brain variations at the network level and applied it specifically to schizophrenia (SZ) disorder. The analysis is based on the application of independent component analysis (ICA) over the subject's dimension of the FC matrices, obtained from resting-state functional magnetic resonance imaging (rs-fMRI). The dataset included 27 SZ people and 27 completely matched healthy controls (HC). This hypothesis-free approach led to the finding of three brain subnetworks significantly discriminating SZ from HC. The area associated with these subnetworks mostly covers regions in visual, ventral attention, and somatomotor areas, which are in line with previous studies. Moreover, from the graph perspective, significant differences were observed between SZ and HC for these subnetworks, while there was no significant difference when the same parameters (path length, network strength, global/local efficiency, and clustering coefficient) across the same limited data were calculated for the whole brain network. The increased sensitivity of those subnetworks to SZ-induced alterations of connectivity suggested whether an individual scoring method based on their connectivity values can be applied to classify subjects. A simple scoring classifier was then suggested based on two of these subnetworks and resulted in acceptable sensitivity and specificity with an area under the ROC curve of 77.5%. The third subnetwork was found to be a less specific building block (module) for describing SZ alterations. It projected a wider range of inter-individual variations and, therefore, had a lower chance to be considered as a SZ biomarker. These findings confirmed that investigating brain variations from a modular viewpoint can help to find subnetworks that are more sensitive to SZ-induced alterations. Altogether, our study results illustrated the developed method's ability to systematically find brain alterations caused by SZ disorder from a network perspective.

## 1. Introduction

Schizophrenia (SZ) is a complex challenging mental disorder resulting in significant functional, behavioral, and cognitive impairments (Friston and Frith, 1995; Palmer et al., 1997; Green et al., 2000; Luck and Gold, 2008; Sponheim et al., 2010; Rubinov and Bullmore, 2013). This disorder, which affects about 0.45% of the adult population worldwide^1^ (GBD 2016 Disease Injury Incidence Prevalence Collaborators, 2017), is rooted in a combination of genetic and environmental factors; although the exact causes are still unclear. SZ is known as a brain disconnectivity syndrome (Friston and Frith, 1995; Yu et al., 2012; A Ure et al., 2018; McNabb et al., 2018; Li S. et al., 2019), pointing to abnormal interaction between critical areas of the brain. Focusing on connectivity abnormalities, functional magnetic resonance imaging (fMRI) has been increasingly used to study brain dysfunctions in individuals living with SZ (Yu et al., 2012; Dong et al., 2018; Adhikari et al., 2019). Several studies have found evidence for altered resting-state functional connectivity (FC) in different brain regions of SZ people compared to healthy control (HC) subjects (Yu et al., 2012; Karbasforoushan and Woodward, 2013; Dong et al., 2018; Zhuo et al., 2018; Bulbul et al., 2022; Cai et al., 2022). Basically, our various brain functions are generated by its interactive connections. Therefore, exploring brain variations in disorders at the network level can be more helpful in understanding brain functionality alterations in SZ people.

Exploring brain abnormalities in SZ individuals compared to HC can be investigated by a variety of methods at the voxel or regional level. In these studies, regions of interest (ROI) are usually identified through seed-based analysis (Zhou et al., 2007; Whitfield-Gabrieli et al., 2009; Salvador et al., 2010; Woodward et al., 2011; Zhuo et al., 2018; Li S. et al., 2019; Li X.-B. et al., 2019; Gong et al., 2020; Ahmad et al., 2023) or independent component analysis (ICA) (Calhoun and Adali, 2012; Anderson and Cohen, 2013; Lottman et al., 2019; Salman et al., 2019; Forlim et al., 2020), and then, the functional activity of desired regions or connectivity strength of each voxel/region pair is compared across SZ and HC (Camchong et al., 2011; Wolf et al., 2011; Mingoia et al., 2012; Yu et al., 2012; Karbasforoushan and Woodward, 2013; Li S. et al., 2019). Moreover, several connectivity parameters of graph theory have been evaluated for the whole brain network, indicating a significant increase in path length in SZ people and a significant decrease in nodal degree, functional connectivity strength, global efficiency, small-worldness, etc. (Liu et al., 2008; Lynall et al., 2010; Micheloyannis, 2012; Anderson and Cohen, 2013; Hadley et al., 2016; Xiang et al., 2020). Overall, the abnormal areas are mostly sought through primary hypotheses or by an overall (random) search of the whole brain. The latter depends on the search algorithm, and the former is limited by the accuracy of prior knowledge.

Regardless of the search algorithm, the findings of previous methods usually report the alterations in some brain regions or some scattered connections, rather than describing them in the form of modulating networks. Such modulating networks have been recently introduced in a study (Keyvanfard et al., 2020), where a blind ICA approach discovered the building blocks (units) of inter-individual brain variations at the network level. It has been shown that each derived building block may participate in the modulation of several brain functions related to inter-subject variations. Introducing more subjects with new variations (caused by the disorder) is expected to result in the formation of new components encompassing the brain variations related to the SZ.

Therefore, the main purpose of this study was to investigate the usability of the previously proposed approach (Keyvanfard et al., 2020) in determining units of inter-group variations between SZ and HC. In the current study, we modify the previously developed algorithm and investigate the alteration of brain connections (subnetworks) due to including the SZ group in addition to the HC group. This modification consists of two steps: improving the component reproducibility and providing a new method of edge pruning (and therefore the number of pruned edges will not be similar for all subnetworks). We also examined how similar the obtained subnetworks are to the well-known resting-state networks (RSNs) (Smith et al., 2009). Through this systematic method of the developed approach, we expect to obtain brain subnetworks that are more sensitive to connection alterations due to SZ. This higher sensitivity could potentially help to introduce new biomarkers or efficient classifiers.

## 2. Materials and methods

### 2.1. Participants, data acquisition, and preparation

In this study, we retrospectively used the resting-state functional MRI (rs-fMRI) data of an SZ group of 27 subjects (mean age, 41.9±9.6) and the completely matched control group of 27 healthy individuals [mean age, 35±6.8; datasets are publicly available on the Zenodo platform (Vohryzek et al., 2020)]. The individuals in the SZ group had been recruited from the Service of General Psychiatry at the Lausanne University Hospital. They had met DSM-IV criteria for SZ and schizoaffective disorders (American Psychiatric Association, 2000). Healthy controls had been recruited through advertisement and assessed with the Diagnostic Interview for Genetic Studies (Preisig et al., 1999). Subjects with major mood, psychotic, or substance-use disorders and having a first-degree relative with a psychotic disorder had been excluded. Moreover, a history of neurological diseases was an exclusion criterion for all subjects. The informed written consent had been obtained for all subjects according to the Ethics Committee of Clinical Research of the Faculty of Biology and Medicine, University of Lausanne, Switzerland (#82/14, #382/11, #26.4.2005). For each participant, two types of MR imaging including rs-fMRI and T1-weighted had been acquired using a 3T Siemens Trio Scanner equipped with a 32-channel head coil.

The magnetization-prepared rapid acquisition gradient echo (MPRAGE) sequence had been applied for T1-weighted imaging with a resolution of 1 × 1 × 1.2 mm^3^ and TI/TE/TR = 900/2.98/2,300 ms. Each rs-fMRI scan had a duration of 8 min with a 3.3 mm isotropic voxel size and TE/TR = 30/1,920 ms. The performed data preprocessing included the exclusion of the first four time points of signal, regressing out of physiological signal (white-matter and cerebrospinal fluid), motion correction, physiological noise correction, spatial smoothing, bandpass filtering, and linear registration to the T1-weighted image.

Employing the Desikan Killiany atlas (Desikan et al., 2006) and extra parcellation of the cortical surface described in Cammoun et al. (2012), the gray matter of each subject in MPRAGE volume had been partitioned into 129 cortical regions of interest (ROI) including 114 cortical ROIs and 14 subcortical nuclei plus the brain stem. These brain regions had been used to estimate the functional connectivity matrices based on the Pearson correlation between individual brain regions' time courses. Finally, one functional connectivity matrix with the dimension *K*×*K* (with *K* = 129 number of the brain parcels) had been constructed for each participant [please refer to Vohryzek et al. (2020) for detailed information.] Here, to consider the connections' strength, the absolute value of the Pearson correlation in the functional connectivity matrix was utilized. The functional connectivity matrices were then evaluated for normality through the Shapiro–Wilk test (Shapiro et al., 1968). Their *p*-value > 0.48 (>>0.05) indicated that they follow a normal distribution.

### 2.2. PCA and ICA

The lower triangular elements of the FC matrix of each subject were considered for constructing the feature matrix. The elements were reshaped into a one-dimensional vector of the size $\frac{K\left(K-1\right)}{2}$, where *K* = 129 is the number of parcels. Afterward, FC vectors of all subjects were stacked to form the *X* matrix. Thus, *X* had *N* rows; the number of subjects and $\frac{K\left(K-1\right)}{2}$ columns. Principal component analysis (PCA) was then performed on the feature matrices along the subject dimension and 90% of data variance was preserved. *X*′ is the new representation of the original feature matrix after applying PCA. The next step is source extraction from the *X*′ matrix using the ICA approach as in Eq (1).


(1) $$\begin{array}{l} {\text{X}}^{\text{′}}=\text{A}\;\times \;\text{S} \end{array}$$

where ***A*** is the mixing matrix and ***S*** is the independent source. ICA was performed based on the Infomax (Bell and Sejnowski, 1995) algorithm. Each row in ***S*** is considered one component, whose values determine the contribution of edges in that component. We call these values the “ICA value” of the edges in the rest of this study. A few of these components have a major role in forming the feature matrix *X*′. Selecting the important components among all of them requires an algorithm to assess the reproducibility of the components during different runs. Ranking and Averaging Independent Component Analysis by Reproducibility (RAICAR) (Yang et al., 2008) had been previously employed (Keyvanfard et al., 2020) to avoid run-to-run variability of components ordering and identify reproducible components across 100 ICA runs. Nevertheless, the edge value of obtained (ordered) components in RAICAR was averaged and therefore varied during different runs, and it resulted in dissimilar final subnetworks in different runs. To overcome this limitation, we modified the RAICAR algorithm by considering the correlation values as well as the number of similar components, so that every time, it resulted in the same components with no need to average or perform any kind of manipulation of the edge values. Details are discussed in section 1 of the Supplementary material.

### 2.3. Edge pruning

The components derived from ICA consist of all brain connections with different weights (the ICA values). Edge pruning is, therefore, required in order to keep only the important connections. This had been previously performed through their *z*-score values (normalization of connections of each component by subtracting the mean value of that component and dividing by its standard deviation) and thresholding them (Keyvanfard et al., 2020). This would result, however, in having a similar number of connections remaining in each component (due to their normal distribution). Here, we modified the pruning algorithm by revisiting the definition of importance for each edge. In the previous criterion (based on *z*-score), the weight values of edges specified their importance level. Here, their effect on the reversibility of the ICA procedure was replaced instead of that criterion. We, therefore, developed a new algorithm (detailed in section 2 of the Supplementary material) to calculate the contribution of each edge to the reconstruction of the original functional connectivity matrices. The edges with maximum effects on the reversibility of the ICA procedure were kept, and the others were replaced by zero. The component after edge pruning will be hereafter referred to as a “subnetwork.”

**Figure 1:**
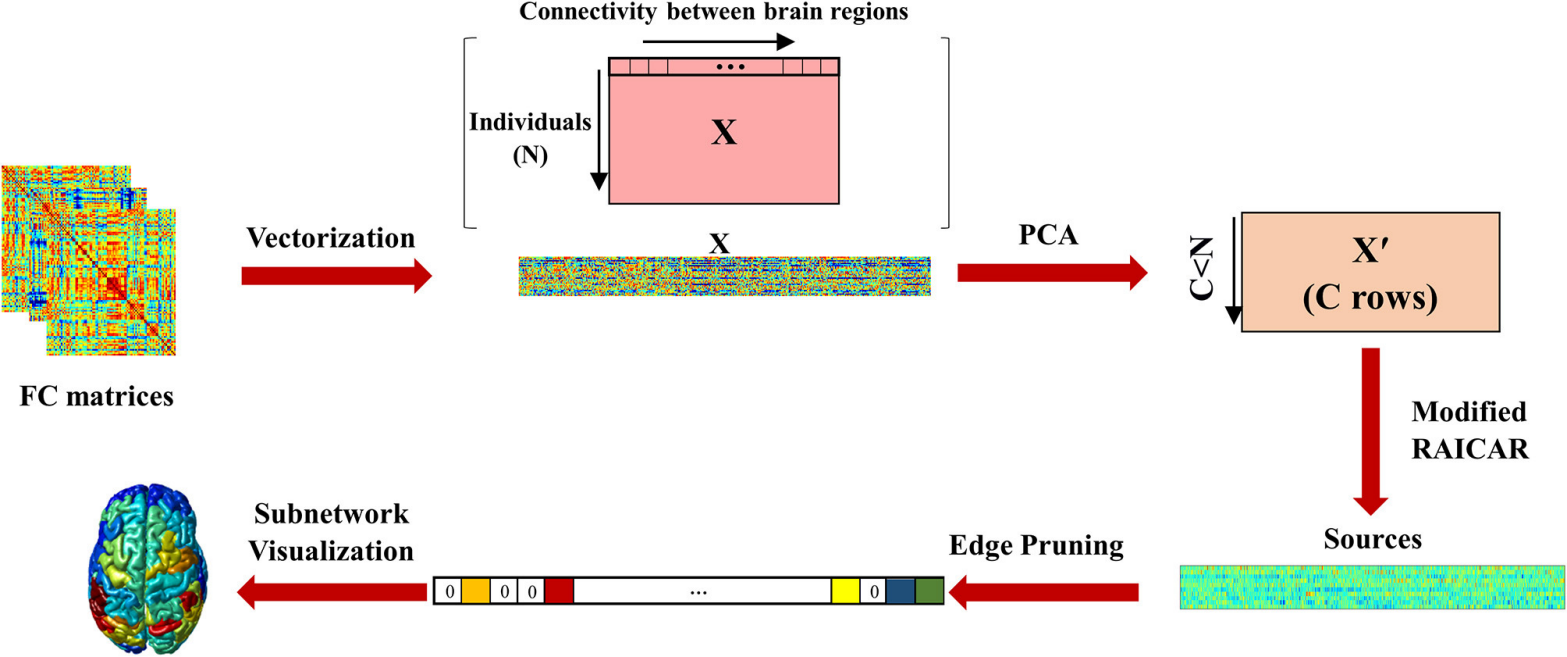
The proposed pipeline. The functional connectivity matrices of all participants were vectorized and stacked on top of each other. The size of this matrix was $N\times \left(\frac{K\left(K-1\right)}{2}\right)$. PCA was performed to reduce information redundancy. The reduced feature matrix had *C* < *N* rows. The modified RAICAR algorithm based on Infomax was then applied. Next, edge pruning was performed to select the most important edges in each component (zero edges show pruned connections and the colors indicate different ICA values of edges). Finally, cortical surface maps of functional subnetworks were constructed based on their normalized nodal strengths.

The pipeline of the proposed algorithm is shown in Figure 1.

### 2.4. Statistical analysis

The subnetworks should be statistically evaluated to determine significantly different subnetworks between the HC and SZ groups. To this end, each individual was projected on a vector, which was defined by two different methods as described below. The statistical analysis was then performed on the projected values.

A) In this viewpoint, only the location of preserved connections (rather than the ICA values) was considered on each subnetwork. For the subnetwork *i*: first, the location of the selected edges was determined. Second, the mean weight of the original functional connectivity values of the selected edges in the HC and SZ groups was calculated. Third, the difference between these two mean vectors was calculated (*Diff(i)*). Then, for each subject in the two groups, the original functional connectivity vector of selected connections was projected on the difference vector (through the inner product) as follows:

***for subnetwork***(***i***)**:**

***Diff***(***i***)**=**|***V***_***HC***_(***i***)**−*V***_***SZ***_(***i***)|,
***for subject***(***j***)**:**

(2) $$\begin{array}{l} \begin{array}{l} \text{A}-\text{Pr}{\text{j}}_{\text{HC}}(\text{i},\text{j})=\;\langle \text{Diff}(\text{i})\;,\;\text{F}{\text{C}}_{\text{HC}}(\text{i},\text{j})\rangle \end{array}\\ \;\begin{array}{l} \text{A}-\text{Pr}{\text{j}}_{\text{SZ}}(\text{i},\text{j})=\langle \text{Diff}(\text{i})\;,\;\text{F}{\text{C}}_{\text{SZ}}(\text{i},\text{j})\rangle \end{array} \end{array}$$

where *V* is the average vector of FC values of subnetwork *i*, FC (*i*,*j*) is the functional connectivity vector of individual *j* at the location of selected connections in subnetwork *i*, and 〈*x, y*〉 illustrates the inner product of *x* and *y* vectors. Finally, a two-sample *t*-test was applied between the obtained projected vectors, ***A***−***Prj***_***HC***_(***i***) **and** ***A***−***Prj***_***SZ***_(***i*** ).

B) Since some selected locations were common among different subnetworks, in this comparison, we were interested in giving value to the degree of contribution of each edge. In other words, the ICA values of the selected edges in the subnetworks were considered. The functional connectivity weights of each individual were projected on the subnetworks through the inner product. Statistical analysis was then applied between these projected values of the two groups (***B***−***Prj***_***HC***_**,*B***−***Prj***_***SZ***_**)**. Here, functional connectivity averaging is no longer performed over individuals and is, therefore, expected to be more robust against inter-individual variations within a group.

***for component***(***i***)***and subject***(***j***)**:**


(3) $$\begin{array}{l} \begin{array}{l} \text{B}-\text{Pr}{\text{j}}_{\text{HC}}(\text{i},\text{j})=\;\langle \text{Comp}(\text{i})\;,\;\text{F}{\text{C}}_{\text{HC}}(\text{j})\rangle \; \end{array}\\ \;\begin{array}{l} \text{B}-\text{Pr}{\text{j}}_{\text{SZ}}(\text{i},\text{j})=\langle \text{Comp}(\text{i})\;,\;\text{F}{\text{C}}_{\text{SZ}}(\text{j})\rangle \end{array} \end{array}$$

The Bonferroni correction (Benjamini and Hochberg, 1995) for multiple comparisons was employed for both parts, A and B, and the *p*-value < 0.005 was considered statistically significant.

Furthermore, the practical significance of the outcomes was evaluated through the calculation of the effect size. Cohen's *d*, the most common measurement method of effect size was used, where the mean difference between the two groups is divided by the pooled standard deviation.

### 2.5. Components evaluation

The distribution of connectivity values for the subnetworks that significantly differentiated the SZ group from HC, and called “significant subnetworks” hereafter, was compared with the well-known RSNs introduced by Yeo et al. (2011). Yeo's atlas includes seven RSNs: visual (VIS), somatomotor (SM), dorsal attention (DA), ventral attention (VA), frontoparietal (FPN), default mode network (DMN), and limbic (Limb) functional systems. These RSNs are illustrated in Supplementary Figure 2. The overlap between the subnetworks and the seven RSNs was determined by computing nodal strengths summation of the common nodes between the RSNs and the obtained subnetworks (Keyvanfard et al., 2020). The detail can be found in section 4 of the Supplementary material.

A non-parametric permutation test was used to statistically evaluate the overlap percentage between the subnetworks and the RSNs. To this end, connection weights were randomly assigned to each subnetwork and the overlap percentage with the RSNs was recalculated. This procedure was repeated 1,000 times. The *p*-value was then computed as the number of times that the newly obtained overlap percentage exceeded the test statistic obtained from the original data, divided by the number of permutations. The significance level was set to 0.005.

### 2.6. Graph parameters

We performed graph theory analysis to examine the connectivity characteristics of the whole brain network as well as the obtained subnetworks. The whole brain network analysis was conducted on weighted and fully connected FC matrices of each individual. In other words, no thresholding and binarization were applied to the adjacency matrices. The five common topological measures were computed: shortest path length, network strength, global and local efficiency, and clustering coefficient, and then, *t*-test statistical analysis was applied between these graph measures of the two groups, HC and SZ.

In the next step, the graph theory analysis was performed on the obtained subnetworks. For each individual, the weighted subnetwork graph was constructed based on the original functional connectivity values at the location of the selected edges. The same five graph metrics were calculated and then statistically analyzed to determine significant differences between SZ and HC groups.

All graph theoretic measures for the weighted graphs were computed with the Brain Connectivity Toolbox (Rubinov and Sporns, 2010). In this section, a *p*-value < 0.05 was considered to indicate statistical significance.

The graph topological characteristics utilized in this study are described as follows:

The shortest path length of a node pair {*L*_*i, j*_} is the minimum edge weight summation required to link the *i*th and the *j*th node. The average of the shortest path length of the network *L*_*net*_, or characteristic path length, is the mean of the shortest path length between all node pairs *(N)* in the network (Watts and Strogatz, 1998):


(4) $$L_{net}\;=\;\frac{1}{N\text{​}\left(N-1\right)}\sum_{i\neq \;j}^{}{\text{L}}_{ij}$$

Network strength (S) is computed as the average of the nodal strength (defined as the summation of all absolute edge values (***w***_***ij***_) connected to each node) of all nodes in the network (Liu et al., 2017). It is described as follows:


(5) $$\begin{array}{l} \text{S }=\;\frac{1}{\text{N}}\sum_{\text{i}\neq \text{j}}{\text{w}}_{\text{ij}} \end{array}$$

Global efficiency (E_global_) measures the degree of integration of brain networks (Latora and Marchiori, 2001, 2003; Achard and Bullmore, 2007). It is the inverse of the average of the shortest path length between each node pair and is defined as follows:


(6) $${\text{E}}_{\text{global}}\;=\;\frac{1}{\text{N}(\text{N}-1)}{\sum^{\text{​}}}_{\text{i}\neq \text{j}}\frac{1}{\text{min}\{{\text{L}}_{i,j}\}}$$

The local efficiency (*E*_i_*local*_) could be interpreted as how well the nodes in the subgraph *G*_*i*_ exchange information when the *i*th node is removed, revealing the tolerance of the network (Latora and Marchiori, 2001). Its calculation is as follows:


(7) $$\begin{array}{l} {\text{E}}_{{\text{i}}_{\text{local}}}\;=\;\frac{1}{{\text{N}}_{{\text{G}}_{\text{i}}}\left({\text{N}}_{{\text{G}}_{\text{i}}}-1\right)}\sum_{\text{i}\neq j}\frac{1}{\min\left\{{\text{L}}_{\text{i},j}\right\}} \end{array}$$

The absolute clustering coefficient of a node (*C*_*i*_) in a weighted graph is the ratio of the sum of triangle intensities (***w***_***ij***_) to the number of all possible connections in the subgraph *G*_*i*_ including *k* nodes (Onnela et al., 2005):


(8) $$\begin{array}{l} {\text{C}}_{\text{i}}\;=\;\frac{{\text{E}}_{\text{i}}}{\frac{{\text{k}}_{\text{i}}\left({\text{k}}_{\text{i}}-1\right)}{2}}\;=\;\frac{2}{{\text{k}}_{\text{i}}\left({\text{k}}_{\text{i}}-1\right)}\;\sum_{\text{j},k}{\left({\text{w}}_{\text{ij}},\;{\text{w}}_{\text{i}k},{\text{w}}_{\text{j}k}\right)}^{\frac{1}{3}} \end{array}$$

The clustering coefficient of a network *C*_net_ is then derived by averaging the clustering coefficients of all nodes within the network.


(9) $$\begin{array}{l} {\text{C}}_{\text{net}}\;=\;\frac{1}{\text{N}}\sum_{\text{i}\in G}{\text{C}}_{\text{i}} \end{array}$$

The entire analysis of this study was performed using MATLAB version 2021a.

## 3. Results

Applying the developed algorithm on the concatenated connectivity matrix of both HC and SZ groups led to obtaining brain subnetworks. Considering the robustness of the components (Keyvanfard et al., 2020) and the connectedness of the areas, only the first eight subnetworks were studied in this study (see Discussion for more details). The edge pruning step was performed with a threshold of 0.2, which resulted in more than 30% of the edges remaining in the subnetworks.

The order of the input data was randomly changed 10 times, and the developed algorithm was re-executed. To assess the change in the order of the output components that might occur due to a change in the order of the input data, we used the correlation coefficient as a similarity index to find the corresponding components in every two runs. The modified RAICAR algorithm resulted in the appearance of all components in their stable locations within 10 times implementation. Moreover, evaluation of the ICA value in each component showed that there was no change in the different runs of the algorithm.

### 3.1. Statistical analysis

Based on the two types of scoring for statistical analysis, described in section 2.4, a statistical analysis was performed on each subnetwork and the following outcomes resulted:

A) Considering the location of selected edges in the subnetworks (see Eq. 2), three of eight, #2 (*p* = 0.0025), #5 (*p* = 4 × 10^−5^), and #7 (*p* = 1.7 × 10^−4^) showed a statistically significant difference between HC and SZ people.
B) Subnetwork #5 (*p* = 9 × 10^−4^) and #7 (*p* = 6 × 10^−4^) significantly differentiated SZ from HC using the ICA values in the projected values (described in criteria B, Eq. 3).

The *p*-value of all subnetworks is listed in Table 1. The lower *p*-values of subnetwork #5 and subnetwork #7 in Part A (Table 1) may indicate that they are more specific to the SZ and called the “SZ-specific” subnetworks hereafter. This outcome can be also inferred from *p*-values in Part B (Table 1) in which considering the ICA values resulted in an increased *p*-value (to be not significant anymore) for #2. Moreover, the reported effect sizes in Table 2 support this outcome. Cohen's *d* for #2 in Part A was higher than 0.8 which implied a large impact; however, this value was less than the other two subnetworks. In Part B, the effect size of subnetwork #2 decreased to 0.3, which revealed its almost limited practical application compared to Cohen's *d* > 0.7 in #5 and #7.

**Table 1:** *p*-values of all subnetworks through two types (A and B) of statistical analysis.

**Subnetwork #**	**1**	**2**	**3**	**4**	**5**	**6**	**7**	**8**
*p*-values in criteria A	0.0072	**0.0025**	0.056	0.049	**4** **×10**^**−5**^	0.013	**1.7** **×10**^**−4**^	0.0076
*p*-values in criteria B	0.505	0.203	0.169	0.414	**9 × 10** ^**−4**^	0.286	**6 × 10** ^**−4**^	0.232

The bold *p*-values (< 0.005) are statistically significant subnetworks.

**Table 2:** Effect size of three subnetworks through both statistical analysis, Part A and Part B in section 2.4.

		**HC (mean ±SD)**	**SZ (mean ±SD)**	**Effect size**
Part A	Subnetwork #2	34.5 ± 5.3	29.9 ± 3.7	0.904
	Subnetwork #5	30.976 ± 4.4	25.6 ± 4.7	1.007
	Subnetwork #7	34.5 ± 5.8	28.1 ± 4.6	1.038
Part B	Subnetwork #2	9.4 ± 2.4	8.6 ± 1.7	0.378
	Subnetwork #5	9.7 ± 2.1	8.02 ± 2.2	0.738
	Subnetwork #7	7.1 ± 2.3	4.9 ± 1.4	0.965

It shows the practical significance of the subnetwork #2 is generally less than #5 and #7.

HC, healthy control; SZ, schizophrenia; SD, standard deviation.

### 3.2. The most affected areas/links by SZ

**Figure 2:**
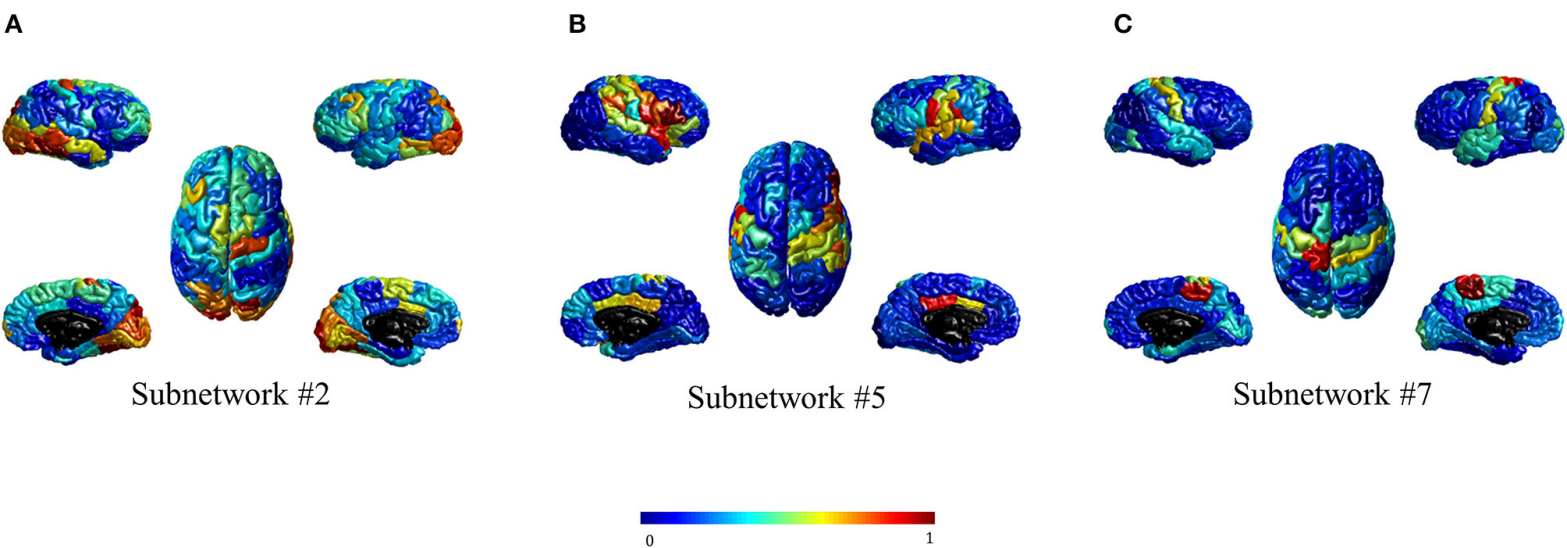
Three brain subnetworks significantly differentiated schizophrenia people from healthy controls. **(A–C)** show subnetworks #2, #5, and #7, respectively. The values are assigned to each region based on the normalized nodal strength. The maps are color-coded in which the larger value is shown in dark red and the smaller one in dark blue.

For visualization purposes, the subnetworks were mapped onto the cortical surface by computing the nodal strength of each region concerning the ICA values. The nodal strength was computed as the summation of the absolute weights of all edges connected to that node. These nodal strength values were then normalized into the range [0–1] for each subnetwork. Figure 2 shows three significant subnetworks.

Figure 2 illustrates that high nodal strength values in subnetwork #2 are mostly observed in regions of the occipital cortex, while for subnetworks #5 and #7, regions in the parietal cortex, and the pre-, post-central gyrus are mostly allocated with high nodal strength, respectively. The visual inspection of subnetwork #2 in Figure 2A with the Yeo atlas (Yeo et al., 2011) indicated that the regions having high nodal strength mostly belong to the visual network. In addition, it seems that the regions in Figures 2B, C can be considered as part of ventral attention (VA) and somatomotor (SM) networks, respectively. This visual inspection was quantified through the computation of their overlap with the RSNs in the Yeo atlas (Yeo et al., 2011) using their nodal strengths. Table 3 represents the overlap percentage of these three subnetworks with the seven RSNs. The bolded values in Table 3 indicate the overlap percentages with *p* < 0.005 in the permutation test.

**Table 3:** Overlap percentage of subnetworks #2, #5, and #7 with the seven well-known RSNs.

**Overlap percentage (%)**	**Vis**	**SM**	**DA**	**VA**	**Limb**	**FP**	**DMN**
Subnetwork #2	**30.9**	9.9	16.1	11.8	7.4	11.7	12.2
Subnetwork #5	1.9	**29.1**	9.5	**40.1**	4.3	5.5	9.6
Subnetwork #7	19.5	**38.8**	9.7	5.8	10.5	7.8	7.8

The items having a p-value < 0.001 in the permutation test are in bold.

Vis, visual; SM, somatomotor; DA, dorsal attention; VA, ventral attention; Limb, limbic; FP, frontoparietal; DMN, default mode network.

**Figure 3:**
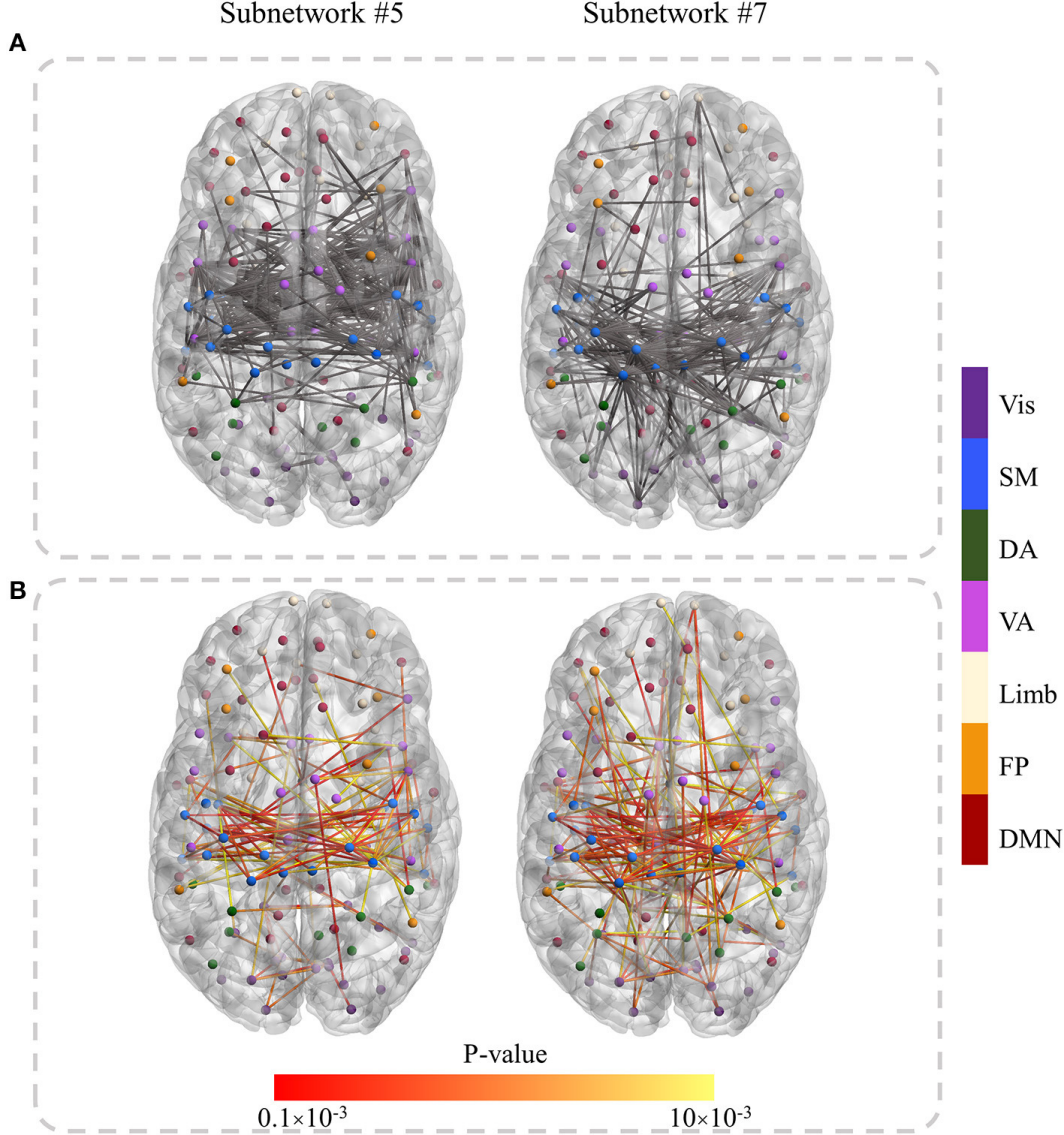
The discriminant connections in subnetworks #5 and #7. **(A)** The connections with absolute ICA values above a threshold (3.5). **(B)** The most discriminant (*p*-value < 0.01) links that were obtained by applying the statistical *t*-test to the original functional connectivity of selected edges in **(A)**. The node color determines the RSN they belong to. The lower *p*-value is shown in red in **(B)**.

To further determine the discriminant connections of a subnetwork, we first selected connections with absolute ICA values >3.5. They are visualized in Figure 3A for the SZ-specific subnetworks (#5 and #7). Then for those selected connections, a *t*-test was performed over the original connectivity values between SZ and HC groups. Connections having *p*-value < 0.01 were visualized in Figure 3B as the most discriminant links (/connections) affected by SZ. It is evident from Figure 3 that the discriminant connections in subnetworks #5 and #7, in particular, link the SM and VA regions in two brain hemispheres.

### 3.3. Graph parameters

**Figure 4:**
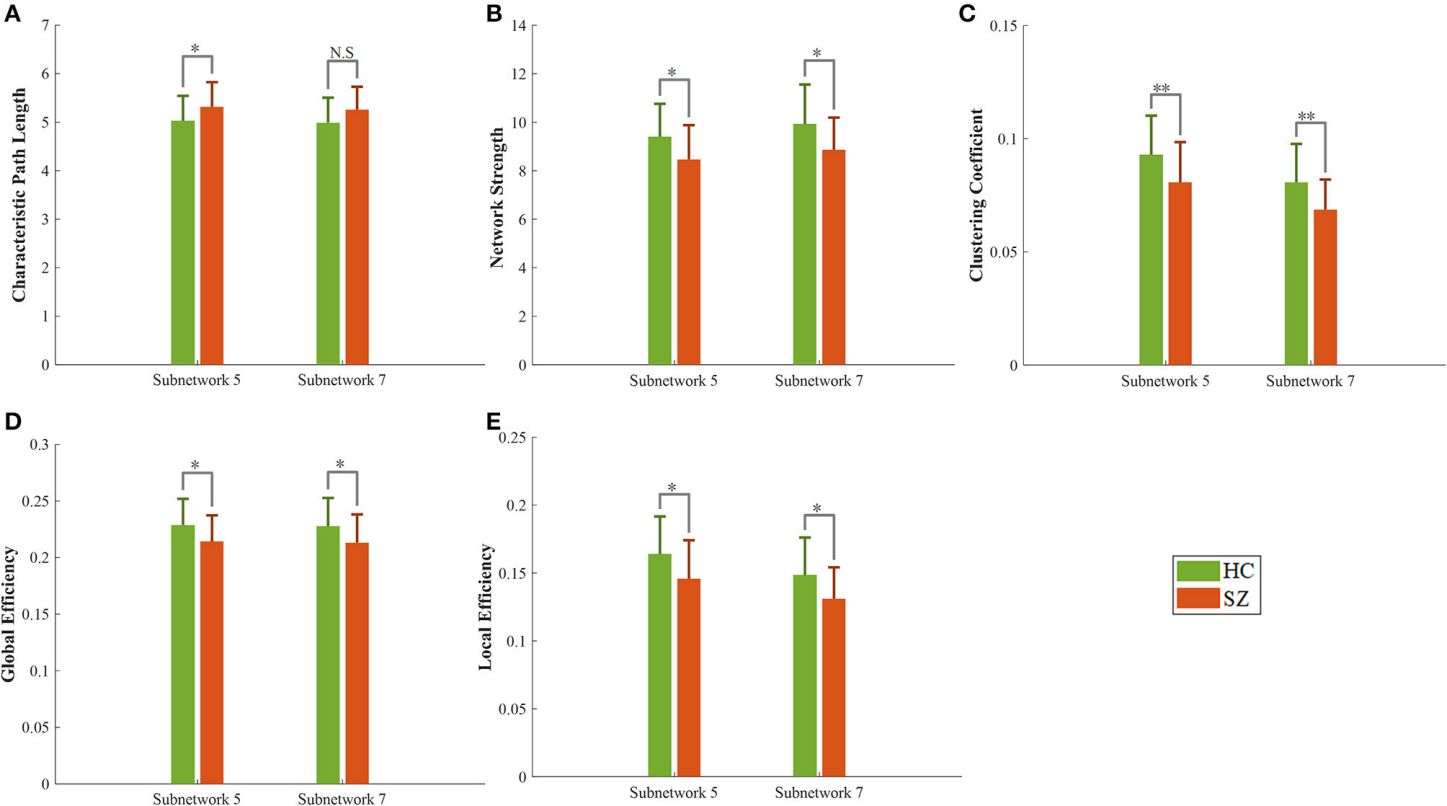
Graph metrics of healthy (green bar) and schizophrenia (red bar) groups in two subnetworks #5 and #7. **(A)** Characteristic path length, **(B)** network strength, **(C)** clustering coefficient, **(D, E)** global and local efficiency. N.S, not significant, *p-value < 0.05, **p-value < 0.01.

There were no significant differences in characteristic path length, network strength, global efficiency, local efficiency, and clustering coefficient of the whole brain network between our limited number of participants in the HC and SZ groups (Table 4). Nevertheless, a significant difference in these five metrics was observed for two subnetworks #5 and #7 over the same dataset (Figure 4 and Table 4). The path length of the SZ group in subnetwork #7, however, was marginally significant. Moreover, the effect size of all metrics of #5 and #7 was >0.5, which implies their acceptable practical impact. All the measured graph metrics of the whole brain and the two subnetworks are given in Table 4. It should be mentioned that the *p*-value in other subnetworks was ≥0.1 for these graph parameters.

**Table 4:** Network topology metrics of two subnetworks, #5 and #7 as well as the whole brain.

	**Subnetwork #5**			**Subnetwork #7**			**Whole Brain**		
	**HC (mean** ±**SD)**	**SZ (mean** ±**SD)**	***p*** **-value/ES**	**HC (mean** ±**SD)**	**SZ (mean** ±**SD)**	***p*** **-value/ES**	**HC (mean** ±**SD)**	**SZ (mean** ±**SD)**	***p*** **-value/ES**
Network strength	9.39 ± 1.35	8.46 ± 1.41	0.016/0.64	9.92 ± 1.62	8.86 ± 1.32	0.011/0.68	30.99 ± 4.6	29.04 ± 4.17	0.13/0.42
Path length	5.03 ± 0.515	5.31 ± 0.51	0.039/−0.54	4.99 ± 0.516	5.26 ± 0.47	0.056/−0.53	3.68 ± 0.338	3.8 ± 0.302	0.18/−0.38
Global efficiency	0.23 ± 0.023	0.21 ± 0.023	0.018/0.61	0.22 ± 0.024	0.21 ± 0.025	0.031/0.60	0.31 ± 0.03	0.29 ± 0.026	0.12/0.43
Local efficiency	0.16 ± 0.027	0.14 ± 0.028	0.014/0.62	0.15 ± 0.027	0.13 ± 0.028	0.021/0.65	0.20 ± 0.036	0.19 ± 0.033	0.16/0.40
Clustering coefficient	0.093 ± 0.017	0.08 ± 0.017	0.006/0.67	0.081 ± 0.016	0.068 ± 0.013	0.008/0.74	0.19 ± 0.037	0.18 ± 0.033	0.16/0.41

A negative effect size in path length indicates its increment in the SZ group.

HC, healthy control; SZ, schizophrenia; SD, standard deviation; ES, effect size.

## 4. Discussion

In the present study, the previously developed algorithm (Keyvanfard et al., 2020) was first modified and then applied to the population of 54 individuals including SZ and HC people. The algorithm output was the components related to the brain variations between individuals (Keyvanfard et al., 2020). The first eight components were considered for further analysis. Considering the selected edges in the subnetworks, three components indicated significant differences between the functional connectivity of SZ and HC groups.

### 4.1. Anatomical distribution of subnetworks

The second subnetwork had a significant and wide overlap (30.9%) with the visual network. Moreover, the statistical analysis of this subnetwork (Part A) indicated a significant difference between the HC and SZ. Therefore, it can be concluded that the visual network variations are somehow related to SZ. It is worth noting that the damaged and reduced functional connectivity of visual resting-state network in SZ people has been previously reported (van de Ven et al., 2017; Arkin et al., 2020; Keyvanfard et al., 2022; Wei et al., 2022). The prior independent reports have also demonstrated abnormalities in different levels of visual processing in individuals living with SZ (Butler et al., 2008; Green et al., 2011; King et al., 2017; Kogata and Iidaka, 2018; Adámek et al., 2022). Furthermore, the recent studies' results have discussed that the visual system has a possible key role in the development of SZ (Benson et al., 2012; Bolding et al., 2014; Morita et al., 2016; Császár et al., 2019).

Subnetwork #5 showed significant overlap (40%) with the ventral attention (VA) network. Its significant difference between the SZ and the control group indicated the functional connectivity of the VA network is disrupted in SZ. Deficits in attentional control are known as a main feature of SZ and a pivotal contributor to cognitive dysfunction (Luck and Gold, 2008; Nuechterlein et al., 2009; Orellana et al., 2012; Arkin et al., 2020). It has also been suggested that complex visual hallucinations reflect dysfunction within and between the attentional networks, leading to the inappropriate interpretation of ambiguous percepts (Shine et al., 2014). The VA network is closely associated with the so-called “salience network.” The salience network has been involved in the pathophysiology of SZ. Its dysfunction results in the incorrect assigning of salience, which can, in turn, lead to the key symptoms of SZ, including delusions (Palaniyappan and Liddle, 2012). Hypoconnectivity within the VA network has been previously reported in several studies (Yan et al., 2012; Wang et al., 2015; Dong et al., 2018; Li S. et al., 2019; Arkin et al., 2020; Keyvanfard et al., 2022).

The somatomotor network regions were partly observed in the third significant subnetwork #7. The nodal strengths in this subnetwork showed a 39% overlap with the somatomotor network. In addition, subnetwork #5 also had a 29% overlap with this RSN (SM). Neurological soft signs such as motor symptoms are common among SZ people [at least one neurological soft sign is detectable in 98% of individuals with SZ (Lane et al., 1996)]. Thus, these signs, while not specific to SZ, are as much a feature of SZ as any other signs or symptoms, and altered FC in somatomotor networks might have been expected (Lane et al., 1996; Shinn et al., 2015). Alteration of the SM areas in SZ has been previously reported in different studies (Zhuo et al., 2014; Dong et al., 2018; Li S. et al., 2019; Liu et al., 2020), and therefore, the emergence of this building block of variations using our developed algorithm is in line with other independent studies.

### 4.2. Specificity of subnetworks

The developed algorithm is designed to decompose the functional connectivity data into components that form the building blocks of group variations. Having a brain disorder is only one of the inter-group variations, and therefore, it is expected that one or more of the obtained components distinguish the SZ group from the healthy one. However, other blocks may relate to other population differences and demographic variables, such as their degree of rationality, gender, and life quality. The developed algorithm was also performed on the healthy group, including 27 subjects, and the similarity of the first eight components was compared with the eight components from 54 individuals (SZ + HC). This comparison indicated high similarity (around 70%) for some subnetworks, such as #1. It suggests that the subnetworks which did not show significant differences between SZ and HC groups can correspond to other inter-individual differences. Here, we aimed to find the SZ-specific subnetworks that discriminate between SZ and healthy groups. No explanation of other obtained subnetworks is, therefore, provided.

It should be also noted that in this algorithm, each component can participate in the modulation of more than one variation, and mutually, each difference in behavioral level may be rooted in a few of these modules (Keyvanfard et al., 2020). We focused on the altered subnetworks in SZ and performed statistical tests based on two different criteria. While the first one (Part A in section 2.4) only considered the location of selected edges in the obtained subnetworks, in the second one (Part B in section 2.4), the importance of each connection in the subnetwork was also considered by using the ICA values. Using the latter test, subnetwork#2 did not display a significant difference between the two groups. It can be inferred that the ICA values in #2 may be affected by various individual variations in the visual network, which inherently exist between people. This component is, therefore, not considered as a SZ-specific subnetwork; a point that can also be concluded from the *p*-values comparison in Part A (Table 1). The *p*-value of #2 in Part A was at least one order of magnitude higher than #5 and #7 but one order of magnitude lower than other (non-significant) subnetworks. Moreover, the effect size of subnetwork #2 was generally less than #5 and #7, confirming the less practical significance of this subnetwork.

### 4.3. Sensitivity to graph analysis

Another interesting finding of this study is related to the topological properties of the obtained subnetworks. The characteristic path length indicates how easily information can be transferred across the network (Rubinov and Sporns, 2010). An increase in this value in the SZ group reveals harder (less flexible) communication across multiple nodes. Network strength shows the overall connectivity through a network and is therefore expected to have lower values in the SZ group. Local efficiency reflects the fault tolerance of the network system or the efficiency of communication between the first neighbors of a node when it is removed. Brain networks with high clustering coefficients and high local efficiency are robust in local information processing even if some neurons are inefficient or damaged (Zhao et al., 2008; Farahani et al., 2019). The low local efficiency and low clustering in SZ suggest that the ventral attention and somatomotor networks in SZ had lower local fault tolerance (i.e., more vulnerable) than the HC group. Reduced local efficiency and clustering coefficients for the whole brain in the SZ group have already been reported in previous fMRI studies (Liu et al., 2008; Lynall et al., 2010). It is worth noting that most previous studies show remarkable changes in these graph metrics of the whole brain in SZ disorder (Liu et al., 2008; Lynall et al., 2010; Micheloyannis, 2012; Xiang et al., 2020). Regardless of the method of graph construction and the amount of change induced by the disease in those metrics, it seems that the change may not be significant on the whole brain for some datasets. However, the significant graph metrics change can be observed in specific subnetworks of the brain. It may be concluded, therefore, that probing these specific brain subnetworks (instead of the whole brain) with those metrics may assist in disorder diagnosis/treatment by elevating the sensitivity.

### 4.4. Feasibility of classification

The higher sensitivity of graph parameters in the SZ-specific subnetworks #5 and #7, compared to the whole brain network, along with their effect size and significant differences they made between SZ and HC, encouraged us to investigate whether the classification of data can be performed through these subnetworks. A simple classifier can be, therefore, suggested to discriminate these two groups' data. The following procedure was performed for both #5 and #7. The mean value and standard deviation of projected functional connectivity (Part B in section 2.4) in the SZ and HC groups were computed. Two z-score values were then assigned to each individual based on his/her obtained values considering the mean and standard deviations calculated for both SZ and HC groups. These values were interpreted as the individual score in the SZ/HC group. The summation of the scores for each individual indicated its class; the HC label was applied if the score value was less than zero, and the SZ label was applied otherwise. To evaluate the classification performance, the metrics including accuracy = (TP + TN)/(TP + TN + FP + FN), precision = TP/(TP + FP), sensitivity = TP/(TP + FN), specificity = TN/(TN + FP), balanced accuracy = (sensitivity + specificity)/2 were used, where TP, TN, FP, and FN represent the numbers of true positive, true negative, false positive, and false negative, respectively. In addition, the area under the receiver operating characteristic (ROC) curve (AUC) was also used to provide a threshold-independent reliability assessment. The AUC value evaluates the overall classification performance of the method. These parameters are stable measures of test performance, and therefore, we also calculated the positive predictive value (PPV) and negative predictive value (NPV). If disease prevalence increases, the positive predictive value will increase and the negative predictive value will decrease (Kuhn and Johnson, 2013; Monaghan et al., 2021; Varoquaux and Colliot, 2022).


(10) $$\begin{array}{l} \text{PPV }=\;\frac{\text{Sensitivity}\;\times \;\text{Prevalence}}{\text{Sensitivity}\;\times \;\text{Prevalence }+\;\left(1-Specificity\right)\;\times \;\left(1-Prevalence\right)}\\ \text{NPV }=\;\frac{\text{Specificity}\;\times \;\left(1-Prevalence\right)}{\left(1-Sensitivity\right)\;\times \;\text{Prevalence }+\;\left(\text{Specificity}\right)\;\times \;\left(1-Prevalence\right)} \end{array}$$

**Figure 5:**
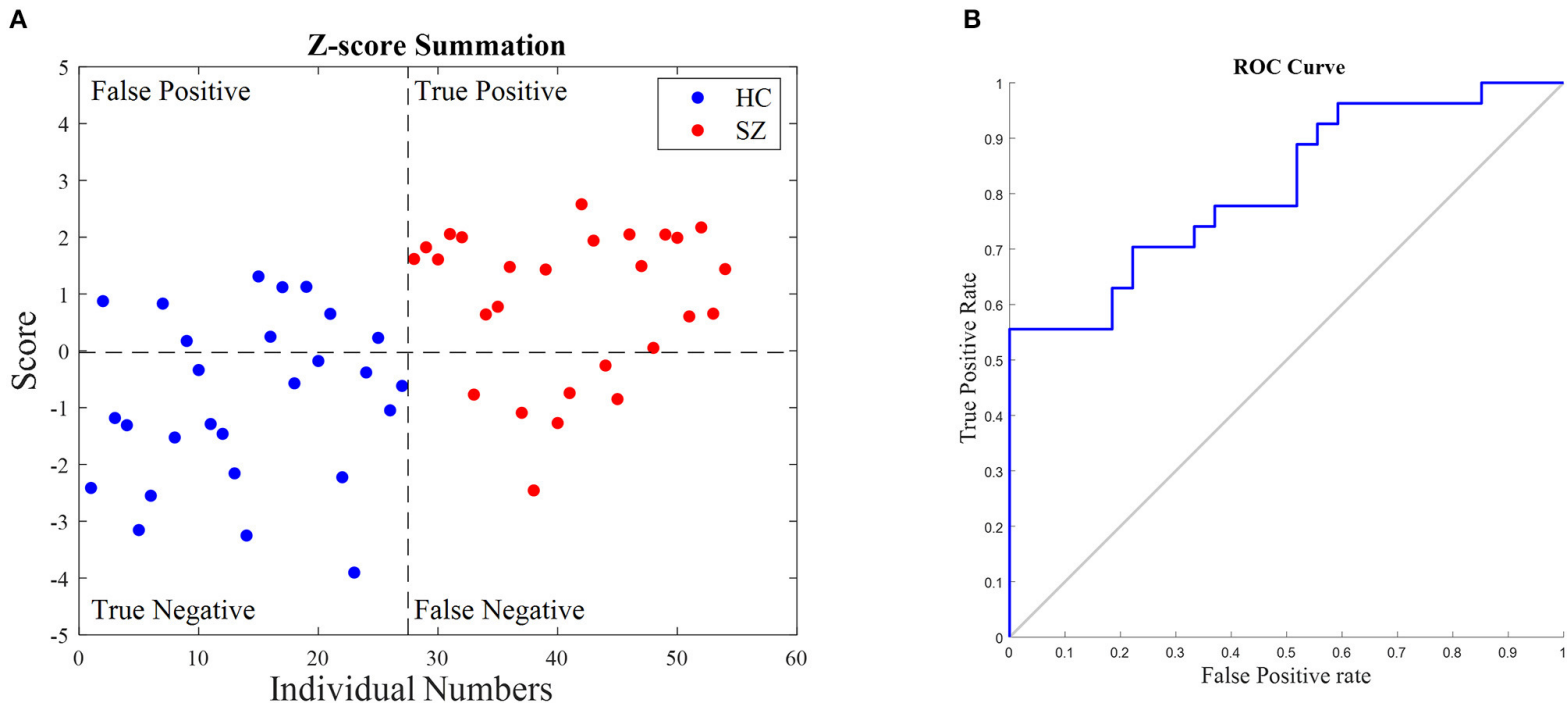
Classification results of schizophrenia people (SZ) and healthy control (HC). **(A)** The summation of their relative *z*-score values in subnetworks #5 and #7 was computed. The horizontal dashed line indicates the classification border, and the vertical one indicates the number of individuals in groups. **(B)** ROC curve of the suggested classifier for all 54 individuals. The area under the curve is 77.5%.

Figure 5A shows the classification result for all 54 individuals. The values above the horizontal dash line indicate the classifier decision as an SZ individual, and below this line correspond to the diagnosis as a healthy person. The ROC curve analysis is shown in Figure 5B. Its AUC is 77.5%. In addition, to evaluate the simple classifier performance, six-fold cross-validation (Efron and Gong, 1983) was used. The dataset was divided into six randomly chosen subsets of equal size (nine subjects). A one-fold was left out to be used as the test subset, and the rest were used for the training subset. Since PCA was utilized in the developed algorithm, to avoid leakage between train and test data, the subnetworks were kept fixed and the training subset indicated the main decision values of each class in the scoring; the mean and the standard deviation.

The six-fold cross-validation was performed 10 times resulting in 60 values for each metric. The performance measures were then calculated as an average value across all folds from all runs, as reported in Table 5. The outcomes of our simple classifier revealed the capability of the obtained subnetworks to distinguish SZ individuals from the HC. Although our main goal in this study is not to propose a classification method, the results showed an acceptable performance compared to the well-known and advanced classifiers, such as neural networks and support vector machines (Shen et al., 2010; Moghimi et al., 2018; Yang et al., 2019; Cai et al., 2020). It should be noted that in contrast to most classification studies where a large number of features are usually used, we assigned only one score to each individual for classification. Nevertheless, the outcome was satisfactory. Furthermore, it can be inferred that our proposed scoring method may be considered as a disorder indicator such as a biomarker. Here, we used six-fold cross-validation to evaluate the classifier for test samples. However, in each run, 45 individuals were utilized as training data and nine samples were considered as tests. Therefore, it can be led to the classifier overfitting. However, to evaluate each classifier's effectiveness, adding new individuals and other datasets is required.

**Table 5:** Classification performance of six-fold cross-validation over 10 runs based on the proposed individual scoring.

**Accuracy**	**Balanced accuracy**	**Precision**	**Sensitivity**	**Specificity**	**PPV**	**NPV**
70.01%	69.35%	69.99%	72.32%	66.38%	63.88%	74.63%

The prevalence of schizophrenia was considered 0.45% for PPV and NPV.

PPV, positive predictive value; NPV, negative predictive value.

### 4.5. Limitation and future study

Applying ICA on the dataset along the subject dimension with M members, in general, results in M components; not all of them are reproducible. In general, applying ICA on a larger dataset leads to more reliable findings. In the preceding version of the algorithm (Keyvanfard et al., 2020), the data of a larger number of healthy subjects (92 individuals) had been utilized and the first 10 components with higher stability in the RAICAR algorithm were considered for further analysis. Here, this research was performed retrospectively, and consequently, the number of available data was limited. In this study, the available data were collected from 27 people with SZ along with their matched group of healthy individuals. We, therefore, chose eight (rather than 10) components based on the components' reproducibility and also connectedness of the corresponding regions in the subnetworks.

The results of this study are, therefore, based on a limited data sample, which may lead to the inadequate generalizability of the results. With a higher number of datasets, we would be able to separate the training and test data and come up with a classifier with a low risk of overfitting. However, the evaluation of the developed algorithm and the scoring classifier for different and large datasets would be our perspective on future research direction. Nevertheless, using this dataset and the proposed blind approach, we found SZ-specific subnetworks that were associated with regions whose changes had been previously reported independently.

The findings of this study suggest that the modular viewpoint for brain activities increases the sensitivity for alteration detection. It further suggests that alterations induced by abnormalities (including SZ) can be revealed in the form of specialized subnetworks with high sensitivity to these alterations. Therefore, future studies will focus on the advanced classification of SZ using the variations of subnetworks as a biomarker. Moreover, tracking the changes in these subnetworks may provide a basis to evaluate the progress/prognosis of the disorder.

## 5. Conclusion

In summary, the discriminating subnetworks related to SZ were obtained via a blind approach in this research. The presented subnetworks mostly covered the visual cortex, ventral attention, and somatomotor networks. The two subnetworks with a large overlap with the ventral attention and the somatomotor networks were more specifically related to SZ and also illustrated the significant graph metric variations. In addition to obtaining the subnetworks that modulate the brain functional connectivity in the disease, the derived ICA values were also found important in finding the discriminating altered links that play a key role in the classification. Using these ICA values can then potentially help in defining a score as a biomarker for SZ. This systematic blind method can be utilized to extract the modulating blocks that describe activity variations in each group of individuals, including those who live with different brain disorders.

## Data availability statement

Publicly available datasets were analyzed in this study. This data can be found at: https://doi.org/10.5281/zenodo.3758534.

## Ethics statement

The studies involving human participants were reviewed and approved by Ethics Committee of Clinical Research of the Faculty of Biology and Medicine, University of Lausanne, Switzerland. The patients/participants provided their written informed consent to participate in this study.

## Author contributions

FK and AN: methodology and investigation. FK: formal analysis, writing—original draft, and writing—review and editing. AN-M: conceptualization, investigation, writing—review and editing, supervision, and project administration. All authors contributed to the article and approved the submitted version.

## Footnotes

^1^WHO report (https://www.who.int/news-room/fact-sheets/detail/schizophrenia).
