# Supplementary material for: Brain subnetworks most sensitive to alterations of functional connectivity in Schizophrenia: a data-driven approach
Source: Front Neuroinform. 2023 May 18;17:1175886. doi: 10.3389/fninf.2023.1175886 (PMC10232974; doi:10.3389/fninf.2023.1175886)
Supplement: Supplementary file 1 [file Data_Sheet_1.docx]

Supplementary Material

**1 Modified RAICAR algorithm**

In the modified RAICAR algorithm, two sections were considered to determine repetitive components. The first one was based on CRCM (cross-realization correlation matrix), the matrix that is constructed through cross-correlation between components *i* and *j* in each repetition. (More details about CRCM can be found in the RAICAR algorithm (Yang *et al.*, 2008). Here, the summation of the correlation coefficient of all elements in the mentioned matrix was used as an index to define similar components in different runs. In the second rule, the repeated components in different runs were compared and matched through their correlation values in CRCM. The number of repetitions was considered an ordering factor. This procedure is briefly shown in Figure S1. The written MATLAB code is publicly available at the following link: <https://github.com/ICA-RAICAR-Pruning/Code>


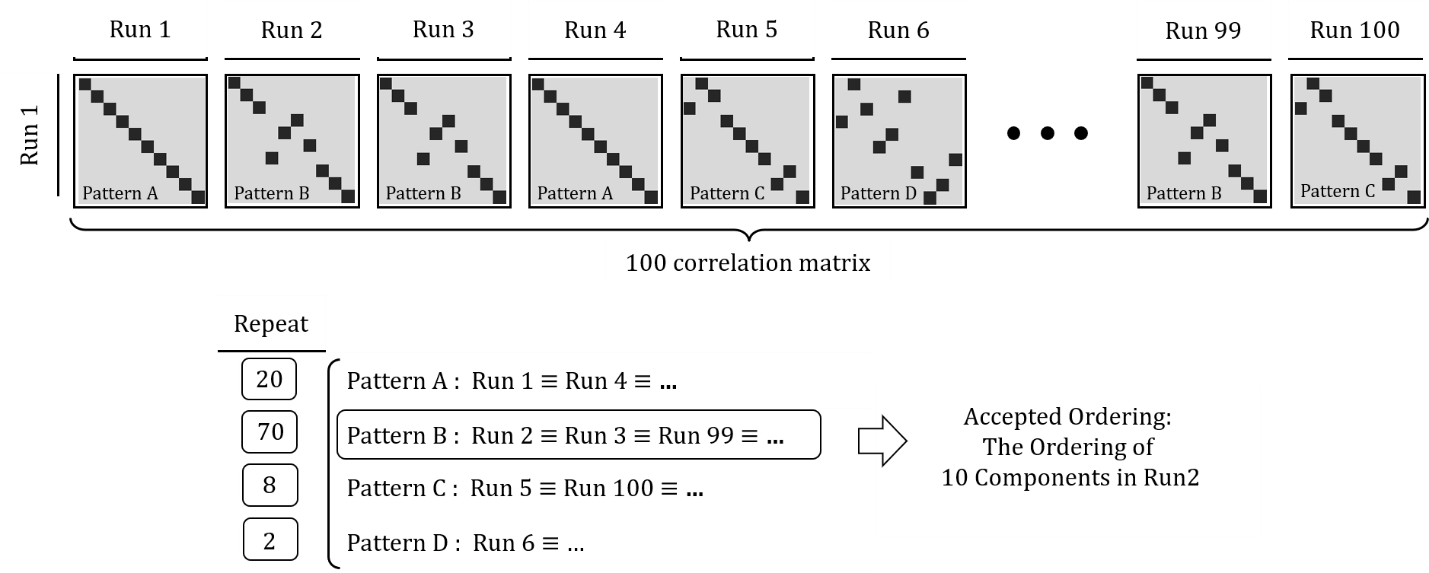


**Supplementary Figure 1.** Component selection in the proposed modified RAICAR. Through 100 runs, every two pairs of correlation matrices were compared. The pattern of correlation matrices was repeated and similar patterns were identified. Then repetition number of each pattern was defined. The final selected pattern was based on maximum repetition through 100 runs.

**2 Edge Pruning**

In the ICA formula (as described in the main text), the effect of each connection (*n*) in source *m*, *S_mn_*, was defined as multiplying every ten values in column m of mixing matrix (*A*) by the *S_mn_*, normalized by the normal value of column *n* in the main feature matrix, *X* (the column influenced by *S_mn_*). The calculated value was then compared by a threshold to define connections with maximum effects. Finally, *S_mn_* was kept if at least one element of the calculated fraction was higher than the threshold value. Therefore, computing all ten elements was not required and in Eq (3), $Effect \left( S_{mn} \right)$ was calculated only based on maximum value of *A_im_*. Formally:

$\left[ \begin{aligned} X_{2n} \\ X_{3n} \\ . \\ . \\ . \\ X_{mn} \end{aligned} \right]=\left[ \begin{aligned} A_{2,1} A_{2,2} \ldots A_{2,m} \\ A_{3,1} A_{3,2} \ldots A_{3,m} \\ . \\ . \\ . \\ A_{m,1} A_{m,2}\ldots A_{m,m} \end{aligned} \right]\times\left[ \begin{aligned} S_{1n} \\ S_{2n} \\ . \\ . \\ . \\ S_{mn} \end{aligned} \right]$ (1)

$Effect \left( S_{mn} \right)=\frac{{max(A}_{2,m},A_{3,m},\ldots,A_{m,m})\times S_{mn}}{\sqrt{{(X}_{2n}^{2}+X_{3n}^{2}+\ldots+X_{10n}^{2})}}$ (2)

$f\left( S_{mn} \right)=\left\{ \begin{aligned} S_{mn} if Effect \left( S_{mn} \right)>thresh \\ 0 if Effect \left( S_{mn} \right)<thresh \end{aligned} \right.$ (3)

It should be noted that the maximum value of each column in *A* is in its first row and these values are in descending order. This causes undesired bias in calculation of $\boldsymbol{Effect}\left( \boldsymbol{S}_{\boldsymbol{mn}} \right)$ which made keeping more edges for the first elements in the first row. To avoid this bias, the first row was discarded in computing $\boldsymbol{Effect}\left( \boldsymbol{S}_{\boldsymbol{mn}} \right)$ in Eq (3). The written MATLAB code of edge pruning is publicly available at the following link: <https://github.com/ICA-RAICAR-Pruning/Code>

**3 RSNs in Yeo’s parcellation**

The color coding of seven well-known Yeo atlas is illustrated in Supplementary Figure 2.


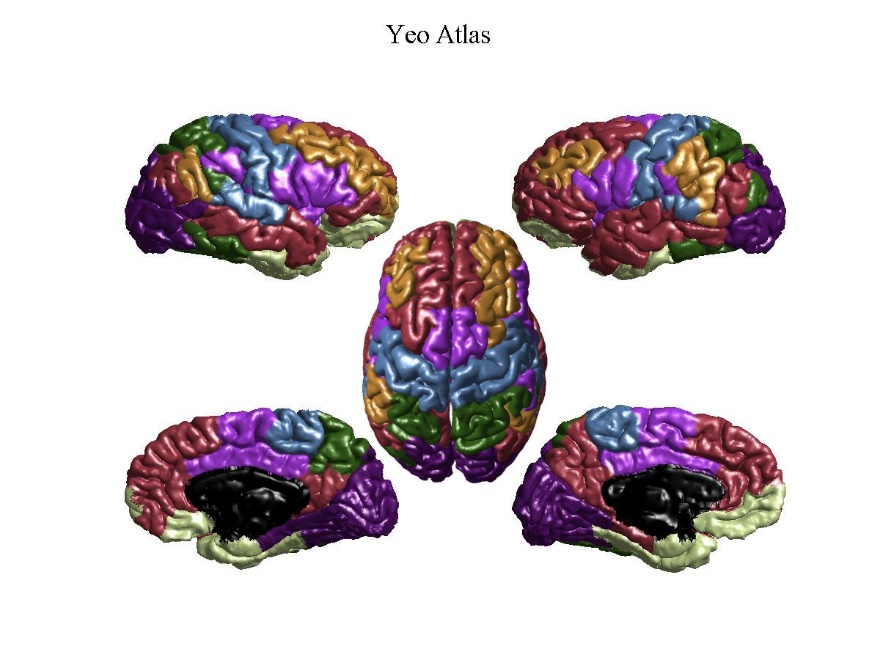


**Supplementary Figure 2** Seven network parcellation of the brain. This parcellation is based on seven networks introduced by Yeo et al., (2011). The assigned color is: Purple (Visual), Blue (Somatomotor), Green (Dorsal attention), Violet (Ventral attention), Cream (Limbic), Orange (Frontoparietal), and Red (Default Mode Network)

**4 Calculation of overlap between subnetworks and RSNs**

Similar to the previously described method (Keyvanfard, Nasiraei-Moghaddam and Hagmann, 2020), to calculate the overlap between obtained subnetworks and RSNs, first, the nodal strength of each node in desired subnetwork was computed. A threshold (here the threshold was set to 0.2) was applied to the normalized nodal strength (*S*) values to discard low ones. The shared nodes between functional subnetwork *i* (*SN(i)*) and RSN *j* was considered and their nodal strength were calculated. These values were normalized by the total strength of the nodes belonging to RSN *j* across all the functional subnetworks. This normalized value was defined as the overlap percentage between each functional subnetwork *i* and each RSN *j*.

$A\left( i,j \right)=\frac{\sum_{k} f(S\left( k \right))}{m} , \mathrm{and}f\left( x \right)=\left\{ \begin{aligned} x x\geq T \\ 0 x<T \end{aligned} \right., k\epsilon\left( SN\left( i \right)\cap RSN\left( j \right) \right), m=\# \left( SN\left( i \right)\cap RSN\left( j \right) \right)$ (4)

$B(i)= \sum_{j=1}^{7} A\left( i,j \right)$ $Op\%\left( i,j \right)=\frac{A(i,j)}{B(i)}\times100$ (5)
